# Analysing Spatio-Temporal Clustering of Meningococcal Meningitis Outbreaks in Niger Reveals Opportunities for Improved Disease Control

**DOI:** 10.1371/journal.pntd.0001577

**Published:** 2012-03-20

**Authors:** Juliette Paireau, Florian Girond, Jean-Marc Collard, Halima B. Maïnassara, Jean-François Jusot

**Affiliations:** 1 Unité d'Epidémiologie des Maladies Emergentes, Institut Pasteur, Paris, France; 2 Unité Epidémiologie/Santé-Environnement-Climat, Centre de Recherche Médicale et Sanitaire (CERMES)/Réseau International des Instituts Pasteur, Niamey, Niger; 3 Unité de Biologie, Centre de Recherche Médicale et Sanitaire (CERMES)/Réseau International des Instituts Pasteur, Niamey, Niger; University of California San Diego School of Medicine, United States of America

## Abstract

**Background:**

Meningococcal meningitis is a major health problem in the “African Meningitis Belt” where recurrent epidemics occur during the hot, dry season. In Niger, a central country belonging to the Meningitis Belt, reported meningitis cases varied between 1,000 and 13,000 from 2003 to 2009, with a case-fatality rate of 5–15%.

**Methodology/Principal Findings:**

In order to gain insight in the epidemiology of meningococcal meningitis in Niger and to improve control strategies, the emergence of the epidemics and their diffusion patterns at a fine spatial scale have been investigated. A statistical analysis of the spatio-temporal distribution of confirmed meningococcal meningitis cases was performed between 2002 and 2009, based on health centre catchment areas (HCCAs) as spatial units. Anselin's local Moran's *I* test for spatial autocorrelation and Kulldorff's spatial scan statistic were used to identify spatial and spatio-temporal clusters of cases. Spatial clusters were detected every year and most frequently occurred within nine southern districts. Clusters most often encompassed few HCCAs within a district, without expanding to the entire district. Besides, strong intra-district heterogeneity and inter-annual variability in the spatio-temporal epidemic patterns were observed. To further investigate the benefit of using a finer spatial scale for surveillance and disease control, we compared timeliness of epidemic detection at the HCCA level versus district level and showed that a decision based on threshold estimated at the HCCA level may lead to earlier detection of outbreaks.

**Conclusions/Significance:**

Our findings provide an evidence-based approach to improve control of meningitis in sub-Saharan Africa. First, they can assist public health authorities in Niger to better adjust allocation of resources (antibiotics, rapid diagnostic tests and medical staff). Then, this spatio-temporal analysis showed that surveillance at a finer spatial scale (HCCA) would be more efficient for public health response: outbreaks would be detected earlier and reactive vaccination would be better targeted.

## Author Summary

Meningococcal meningitis (MM) is an infection of the meninges caused by a bacterium, *Neisseria meningitidis*, transmitted through respiratory and throat secretions. It can cause brain damage and results in death in 5–15% of cases. Large epidemics of MM occur almost every year in sub-Saharan Africa during the hot, dry season. Understanding how epidemics emerge and spread in time and space would help public health authorities to develop more efficient strategies for the prevention and the control of meningitis. We studied the spatio-temporal distribution of MM cases in Niger from 2002 to 2009 at the scale of the health centre catchment areas (HCCAs). We found that spatial clusters of cases most frequently occurred within nine districts out of 42, which can assist public health authorities to better adjust allocation of resources such as antibiotics or rapid diagnostic tests. We also showed that the epidemics break out in different HCCAs from year to year and did not follow a systematic geographical direction. Finally, this analysis showed that surveillance at a finer spatial scale (health centre catchment area rather than district) would be more efficient for public health response: outbreaks would be detected earlier and reactive vaccination would be better targeted.

## Introduction

### Background

Meningococcal meningitis (MM), caused by the bacterium *Neisseria meningitidis* (Nm), is a major health problem in sub-Saharan Africa. The highest incidences of the disease are observed in the so-called “African Meningitis Belt” where annual recurrent epidemics occur during the very hot, dry season [1]. In Niger, reported meningitis cases varied between 1,000 and 13,000 from 2003 to 2009, with case-fatality rates of 5–15%. The factors involved in the spatio-temporal occurrence of meningococcal epidemics are only suspected and still poorly understood.

Surveillance and reactive vaccination are the predominant strategies for managing meningococcal meningitis outbreaks in the Belt, recently completed with a conjugate vaccine to prevent the carriage of Nm serogroup A. In Niger like in most sub-Saharan countries, surveillance is performed at the district level. Quantitative morbidity and mortality data on meningitis are collected within a reporting network managed by the Direction for Statistics, Surveillance and Response to Epidemics (DSSRE) from the Ministry of Public Health. Data from all health care facilities covering the entire Niger population are collected on a weekly basis by the district health authorities, which aggregate and forward their data to the regions and subsequently to the DSSRE. These reported data include all suspected and probable cases, according to the standard clinical definition of meningococcal meningitis [2]: A suspected case is any person with sudden onset of fever (>38.5°C rectal or 38.0°C axillary) and one or more of the following signs: stiff neck, altered consciousness or other meningeal sign; in patients under one year of age, a suspected case occurs when fever is accompanied by a bulging fontanelle. A probable case is defined as a suspected case with turbid CSF or Gram stain showing Gram-negative diplococcus or petechial/purpural rash or ongoing epidemic. Laboratory confirmation of meningitis is not required to report a case. In parallel to this epidemiologic surveillance and in close collaboration with DSSRE, the Centre de Recherche Médicale et Sanitaire (CERMES) is in charge of the national microbiological surveillance of meningitis. The CERMES collects the cerebrospinal fluid (CSF) samples taken from suspected cases of meningitis by health care workers or physicians and carries out the etiological diagnosis (see Methods).

Based on this national surveillance system, the strategy applied in Niger to respond to meningitis outbreaks with limited amounts of available vaccines, is to initiate reactive vaccination in a district once weekly incidence exceeds the epidemic threshold defined by WHO (see [3] and Methods for definitions). Thus, early detection of epidemics is essential for an effective operational response.

### Spatio-temporal analysis

Analysing spatio-temporal patterns of epidemics at a fine geographic scale could lead to a better understanding of the underlying causes of the disease and potential future prediction of outbreaks [4]. One of the techniques to uncover spatial patterns of disease is cluster detection. In epidemiology, a cluster is a number of health events situated close together in space and/or time [5]. Identifying spatial and spatio-temporal clusters of cases could help: (i) to generate new information for further etiologic studies; (ii) to identify risk areas where to focus the surveillance and allocate the resources (antibiotics, rapid diagnostic tests…); (iii) to develop cost-efficient vaccination strategies.

In sub-Saharan Africa, data from national disease notification have already been used at country, regional or district levels to study the geographical and temporal dynamics of epidemics and their correlation with environmental factors [6]–[12]. However, little is known about MM emergence and distribution at a sub-district level. Using a finer spatial scale such as health centre catchment areas (HCCAs) would have several advantages: (i) it would capture heterogeneity in MM incidence at sub-district level; (ii) epidemic thresholds would be studied at a more accurate scale, allowing for a more rapid and targeted public health response; (iii) monitoring of the impact of the intervention would be performed at the same level as the intervention itself.

Therefore, we aimed to investigate the spatio-temporal distribution of MM epidemics in Niger at the health centre catchment area level, to identify the most frequently affected HCCAs requiring a particular attention from public health authorities. The national microbiological surveillance database was used to perform two cluster detection methods in order to uncover spatial and spatio-temporal clustering of MM incidence from July 2002 to June 2009. Then, as a preliminary analysis to a more thorough etiologic study, we searched for ecologic correlation of MM incidence with human density and roads at the HCCA level. Finally, to further investigate the benefit of using a finer spatial scale for surveillance and disease control, we compared timeliness of epidemic detection at the HCCA level versus district level. This paper provides new insights into the spatio-temporal dynamics of MM epidemics and discusses the potential implications of our findings for meningitis control in sub-Saharan Africa.

## Methods

### Data collection and laboratory analyses

The CERMES is the national laboratory in charge of the microbiological surveillance of meningitis in Niger. This surveillance has been reinforced since 2002 [13], [14] by its extension to the whole country (it was only effective in the capital city before 2002) and by the inclusion of a Polymerase Chain Reaction (PCR) assay for etiological diagnosis of meningitis to the DSSRE routine surveillance. CSF samples were collected by health care workers or physicians from suspected cases of acute meningitis. Each CSF was documented with an epidemiological form that included date of sample collection, clinical information and general characteristics about the patient (age, sex, geographic origin such as region, district, HCCA and village). The samples were kept either refrigerated or frozen in health facilities, or inoculated into a trans-Isolate (TI) medium. The more remote health centres sent CSF samples (frozen in a cool box) on a voluntary basis to CERMES by mandated transport companies. Additionally, CERMES carried out active collection of samples twice a day in Niamey, so that the samples remained suitable for culture, and every month within a radius of about 300 kilometres around Niamey, in the regions of Tillabery and Dosso. Etiological diagnosis of MM was carried out by PCR for all CSF as described in [13] and [14] and by culture [15] for suitable CSF received promptly at CERMES (fresh CSF and CSF inoculated into TI medium). Questionnaire data and microbiological results were entered in a database managed by CERMES. The data were used for a retrospective study on meningococcal meningitis cases between July 1, 2002 and June 30, 2009.

### Ethics statement

All data were collected through the national routine surveillance system. Therefore, written consent was not asked and approval from the national ethics committee was not needed. However, patients were informed of the reason why their cerebrospinal fluid was sampled and confidentiality on patients' identity was guaranteed.

### Geographic and demographic data

In 2008, in order to create a digitized National Health Map of Niger, CERMES mapped the country's HCCAs, each of which included all villages served by the same health centre. As projected data were required for the spatial statistics, all analyses were carried out with a projected version of the National Health Map, using the WGS84 – UTM32N projection. The number of inhabitants per village was extracted from the 2001 census database of the Institut National de la Statistique (INS) and an annual population growth rate of 3% was applied. A shapefile of primary roads was retrieved from the HealthMapper application of the World Health Organization (WHO).

### Statistical analyses

#### Detection of spatial clusters and frequency of occurrence

To identify high risk areas on which to concentrate therapeutic and preventive efforts, we searched for annual spatial clusters, defined as groups of MM cases occurring during the same epidemiological year and situated closer together in space than would be expected from the variation in population density and chance fluctuations. Two cluster detection methods were used to uncover spatial clustering of MM incidence at the HCCA level in Niger for each epidemiological year between 2003 and 2009. An epidemiological year *n* was defined as running from July 1 of the year *n−1* to June 30 of the year *n*. The first method was the Anselin's Local Moran's *I* test [4], [16], implemented in the ArcGIS software (version 9.3, ESRI Inc. Redlands, CA), which provided a measure of the spatial autocorrelation for a given HCCA with its neighbours. Spatial clusters of MM cases were identified by mapping the significant *high-high* HCCAs (i.e. high incidence rate HCCA surrounded by high incidence rate HCCAs) and *high-low* HCCAs (i.e. high incidence rate HCCA surrounded by low incidence rate HCCAs). The second method was the Kulldorff's spatial scan statistic [4], [17] implemented in the SaTScan software (version 8.0, Kulldorff and Information Management Services, Inc.), which used a circular moving window to identify single HCCAs or groups of HCCAs of significantly high risk. To determine beforehand the optimal scale for cluster detection by both methods, i.e. the distance where spatial effects were maximized [5], the Global Moran's *I* test was used in ArcGIS and correlograms of *I* against different threshold distances were plotted in R (version 2.9.1, R Development Core Team, R Foundation for Statistical Computing, Vienna, Austria). More details regarding the cluster detection methods can be found in Supporting information (Text S1).

The frequency of spatial cluster occurrence was calculated for each HCCA and each health district, respectively defined as the number of years during which the HCCA contributed to a cluster, and as the number of years during which the district contained at least one HCCA contributing to a cluster. The frequencies were calculated taking into account either (i) clusters detected by one of the two methods (weaker evidence of clustering) or (ii) clusters detected by both methods (stronger evidence of clustering).

#### Detection of spatio-temporal clusters

To analyse the emergence and diffusion patterns of MM cases within each epidemic season, we then searched for spatio-temporal clusters, groups of MM cases situated close together in space and time. The Kulldorff's space-time scan [18] implemented in SaTScan was performed to identify spatio-temporal clusters of maximum one-week duration. More details regarding the method can be found in Supporting information (Text S1).

#### Ecological correlation

To investigate if the highlighted spatio-temporal patterns could be related to host density and movement, correlations of MM incidence at the HCCA level with human density and distance to primary road were explored using Pearson correlation coefficient.

#### Timeliness of epidemic detection

Finally, to evaluate the potential gain in timeliness of epidemic detection at the HCCA level compared to the district-level surveillance, we applied to our HCCA-level data the two thresholds currently used for detection of outbreaks [3]. The alert threshold was defined as 5 cases per 100,000 inhabitants per week for population >30,000 inhabitants or 2 cases in one week for population <30,000 inhabitants. The epidemic threshold was defined as 10 cases per 100,000 inhabitants for population >30,000 inhabitants (under appropriate conditions [3], otherwise 15 per 100,000) or 5 cases in one week for population <30,000 inhabitants. If there was an epidemic in a neighbouring area, the alert threshold became the epidemic threshold.

## Results

### Description of the data

**Figure 1:**
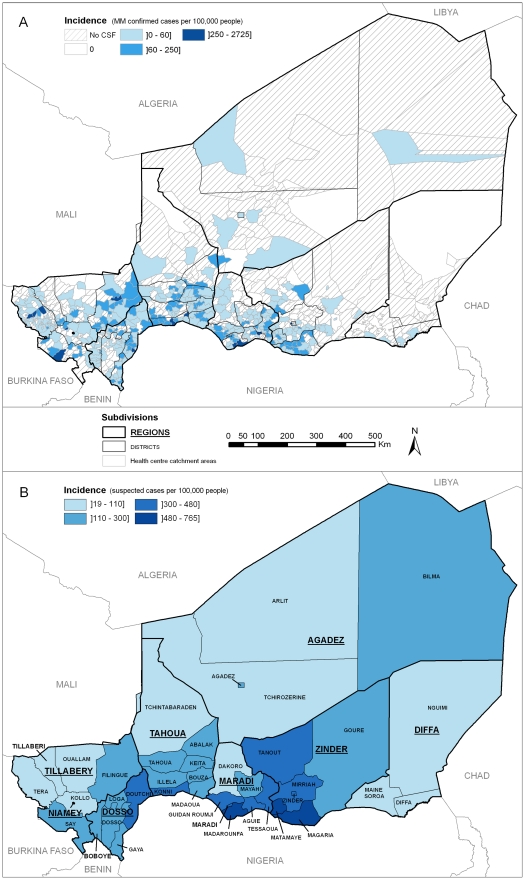
Cumulative incidence rates of meningitis in Niger from July 2002 to June 2009. **A**: Incidence of laboratory-confirmed cases of meningococcal meningitis (MM) at the health centre catchment area level. **B**: Incidence of suspected cases of meningitis reported to the DSSRE at the district level.

From July 1, 2002 to June 30, 2009, a total of 15 801 CSF specimens from meningitis suspected cases were analysed at the CERMES laboratory (table 1). 112 CSF (0.7%) could not be tested (depleted, broken tubes…) and 79 (0.5%) did not give conclusive results because of contamination. Overall, biological specimens originated from 416 (61%) of the 682 HCCAs mapped in 2009. Among these CSF, 6556 (41.5%) were confirmed as bacterial meningitis cases, 82.2% of which were positive for *Neisseria meningitidis*. Serogroup A was the predominant serogroup every year, except in 2006. The mean (SD) age of the MM cases was 9.6 (7.5) years and 58.8% were male. Over the study period, MM cases were detected in 349 HCCAs (51.2%) in all regions of Niger (figure 1), with contrasting incidence rates within districts. The highest incidence rates were found in HCCAs of Niamey, Tillabery, Dosso, Tahoua, Maradi and Zinder regions. As for the temporal distribution, 82.5% of the MM cases occurred from February to April.

**Table 1:** Results of microbiological analyses of cerebrospinal fluid (CSF) samples by epidemiological year.

Year	2003	2004	2005	2006	2007	2008	2009	Total
Collected CSF	2073	1324	1273	3135	1150	2819	4027	15801
**Meningitis positive CSF in number of cases (% of the collected CSF)**								
Positive CSF	945 (45.6)	487(36.8)	370 (29.1)	1336 (42.6)	329 (28.6)	1218 (43.2)	1872 (46.5)	6557 (41.5)
**Bacteria in number of cases (% of the positive results)**								
*N.meningitidis*	793 (83.9)	333 (68.4)	166 (44.9)	1143 (85.6)	137 (41.6)	1060 (87.0)	1756 (93.8)	5388 (82.2)
*S.pneumoniae*	103 (10.9)	123 (25.2)	165 (44.6)	131 (9.8)	128 (38.9)	116 (9.5)	96 (5.1)	862 (13.1)
*H.influenzae*	49 (5.2)	31 (6.4)	39 (10.5)	62 (4.6)	64 (19.5)	42 (3.5)	18 (1.0)	305 (4.7)
Others	0 (0.0)	0 (0.0)	0 (0.0)	0 (0.0)	0 (0.0)	0 (0.0)	2 (0.1)	2 (<0.1)
**Serogroups of ** ***N. _eningitides*** ** in number of cases (% of the meningococci)**								
A	715 (90.1)	280 (84.1)	92 (55.4)	539 (47.2)	119 (86.9)	993 (93.7)	1705 (97.1)	4443 (82.5)
X	3 (0.4)	12 (3.6)	41 (24.7)	559 (48.9)	11 (8.0)	5 (0.5)	10 (0.6)	641 (11.9)
W135	64 (8.1)	31 (9.3)	19 (11.5)	24 (2.1)	5 (3.6)	0 (0.0)	10 (0.6)	153 (2.8)
C	0 (0.0)	1 (0.3)	0 (0.0)	0 (0.0)	0 (0.0)	0 (0.0)	0 (0.0)	1 (<0.1)
Y	4 (0.5)	2 (0.6)	0 (0.0)	1 (0.1)	0 (0.0)	0 (0.0)	1 (<0.1)	8 (0.1)
Undetermined	7 (0.9)	7 (2.1)	14 (8.4)	20 (1.7)	2 (1.5)	62 (5.8)	30 (1.7)	142 (2.6)

### Detection of spatial clusters and frequency of occurrence

**Figure 2:**
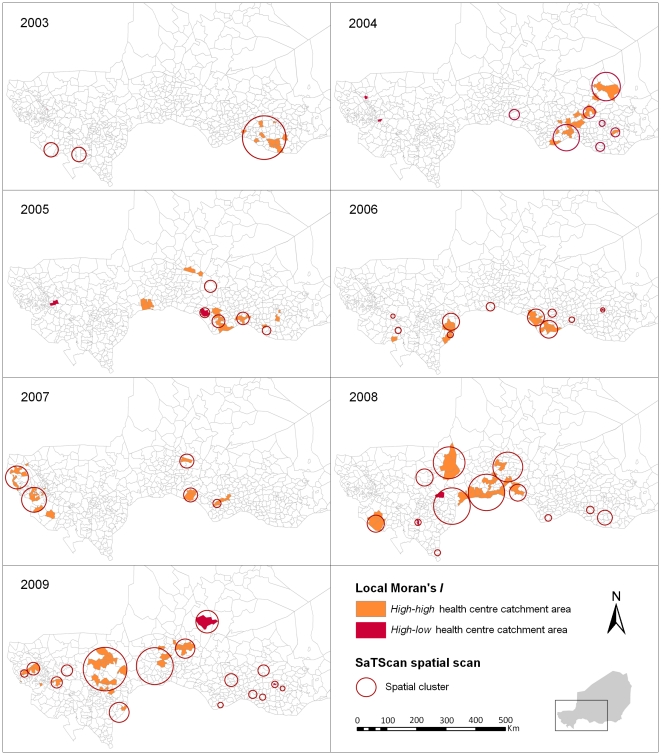
Annual spatial clusters of meningococcal meningitis cases identified in Niger from 2003 to 2009. Each panel shows the results of both methods, the Anselin's Local Moran's *I* test and the Kulldorff's spatial scan statistic, for a single epidemiological year.

Figure 2 depicts for each year the Kulldorff's spatial scan statistic results overlain on the Anselin's Local Moran's *I* results. Over the seven years, the Local Moran's *I* method identified 140 high-risk HCCAs (130 *high-high* and 10 *high-low*), with an annual number ranging from 11 (in 2003 and 2007) to 31 (in 2008 and 2009). The spatial scan method identified 58 significant spatial clusters altogether, with an annual number ranging from 3 (in 2003) to 16 (in 2009). The median number of HCCAs per cluster was 2 (IQ range = 1–5) and the median annual incidence rate of the clusters was 34.9 (IQ range = 20.5–72.3) cases per 100,000. Almost 80% of the high-risk HCCAs identified with the Local Moran's *I* were included in clusters detected by SaTScan and 62% of the SaTScan clusters encompassed *high-high* or *high-low* HCCAs.

**Figure 3:**
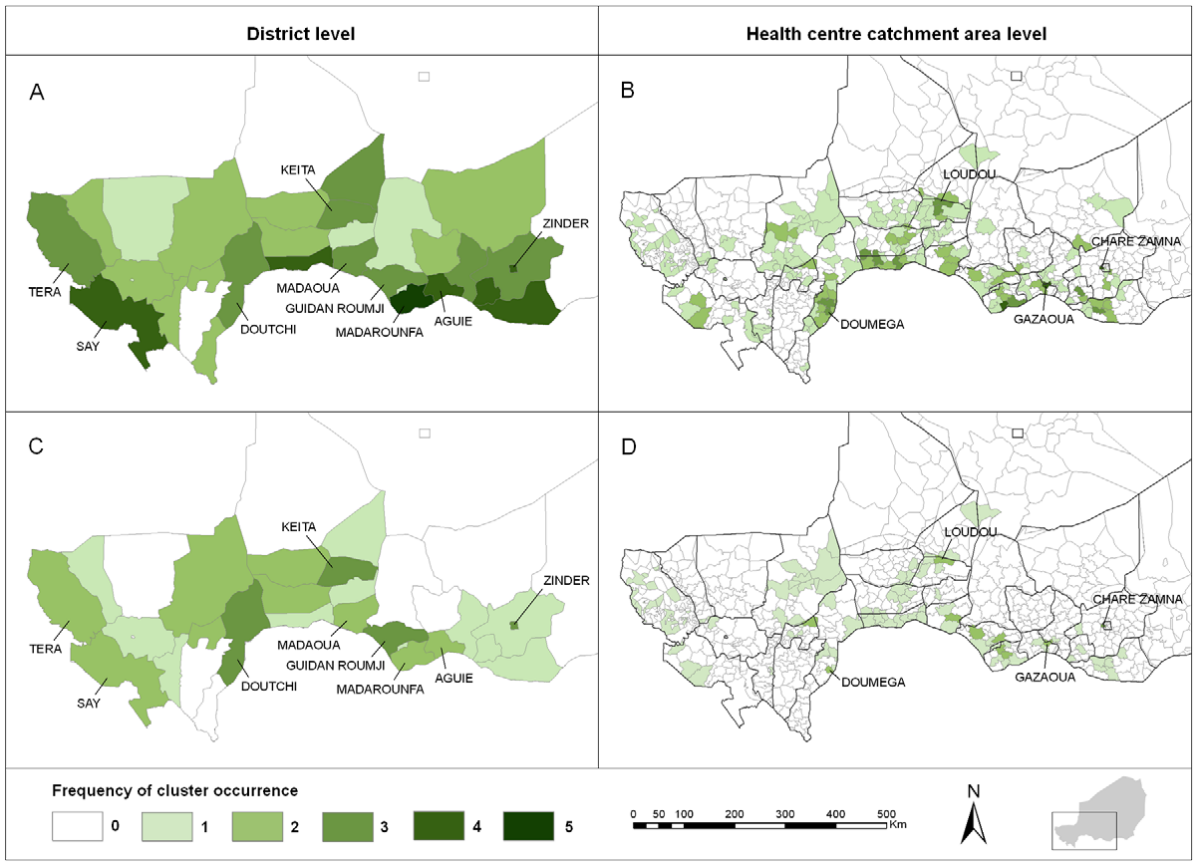
Frequency of cluster occurrence in Niger from 2003 to 2009. **A, B:** Frequencies of occurrence of spatial clusters detected by one of the two methods (Anselin's Local Moran's *I* test or Kulldorff's spatial scan statistic). **C, D:** Frequencies of occurrence of spatial clusters detected by both methods.

Spatial clusters generally occurred in different HCCAs from year to year over the study period, as shown by the low frequencies observed at the HCCA level (figure 3). Among the HCCAs contributing to a cluster at least once over the study period, the median frequency was 1 (range = 1–4) for clusters detected by at least one method, and 1 (range = 1–3) for clusters detected by both methods. Only four HCCAs were detected three or more times by at least one method and two or more times by both methods. They were: Chare Zamna (in Zinder urban community), Gazaoua, Doumega and Loudou.

Spatial clusters most frequently occurred within nine districts out of 42, containing three or more times a cluster detected by at least one method, and two or more times a cluster detected by both methods. These districts were: Tera and Say (bordering Burkina Faso), Keita, Zinder and five districts bordering Nigeria, Doutchi, Madaoua, Guidan Roumji, Madarounfa and Aguie. The median time interval between two clusters occurring in the same district was one year. When a district contained a cluster detected by at least one method, only 13.3% (median) of its HCCAs contributed to that cluster, and 9.7% when a district contained a cluster detected by both methods.

### Detection of spatio-temporal clusters

**Figure 4:**
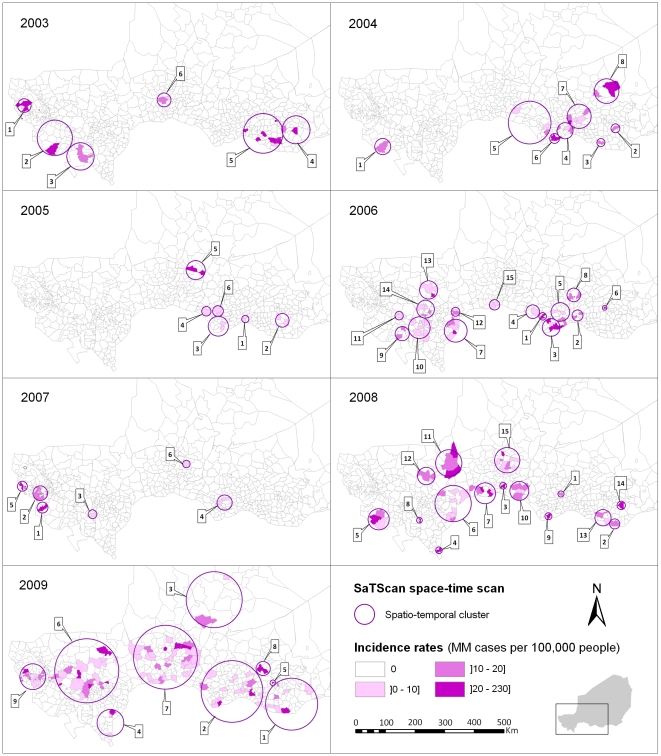
Spatio-temporal clusters of meningococcal meningitis cases identified in Niger from 2003 to 2009. Each panel shows the results of the Kulldorff's space-time scan statistic for a single epidemiological year and the incidence rate observed for each HCCA of a spatio-temporal cluster during the time period associated to that cluster (maximum one week). Spatio-temporal clusters are numbered in chronological order of occurrence.

No systematic spatio-temporal pattern for cluster emergence and epidemic spread was observed within the seven years of the study period. Figure 4 shows the 66 significant spatio-temporal clusters detected with the SaTScan space-time scan (except a 2009 northeast cluster in Dirkou, Bilma district, which is outside the displayed zone) and the incidence rate observed for each HCCA of a spatio-temporal cluster during the time period associated to that cluster. They essentially occurred between February and April, with an additional few at the beginning (November–January) and the end (May) of the epidemics. In 2003, the epidemic could be summarized in two western and eastern poles, with the western pole occurring before the eastern one. In 2004, the first cluster was detected in the west; then all clusters appeared in the east, ending with the northernmost one in Tanout district. In 2005, clusters were detected only in the eastern part. In 2006, two spatio-temporal poles were clearly distinguished, first in the east and then in the west. In 2007, the first three clusters were detected in the west, followed by one in the east, still another one in the west and a final northernmost one in Keita district. In 2008, between the eastern clusters at the beginning and the end of the epidemic season, other clusters essentially appeared in the centre (Tahoua region) and the west (Tillabery and Dosso regions) without a clear order, concluding again the northernmost cluster in Keita district. In 2009, from the first cluster in the east to the final one in the west, clusters appeared in all regions in between, but followed no clear geographical direction.

### Correlation with population density and roads

No significant correlation was found between MM incidence at the HCCA level and human density (r = 0.02) or distance to primary roads (r = −0.07).

### Timeliness of epidemic detection

Between 2003 and 2009, 88 districts crossed the alert threshold. For 42 (47.7%) of them, the alert threshold was crossed earlier (4 weeks early in median) in at least one HCCA of these districts.

Between 2003 and 2009, 46 districts crossed the epidemic threshold. For 15 (32.6%) of them, the epidemic threshold was crossed earlier (3 weeks early in median) in at least one HCCA of these districts.

## Discussion

To our knowledge, this is the first study using health centre catchment areas as spatial units for the spatio-temporal analysis of MM over a whole sub-Saharan country. The study's first finding was the more frequent detection of spatial clusters within nine southern districts, mainly on the southern border with Nigeria. Second, clusters most often encompassed only a few HCCAs within a district, without expanding to the entire district. In addition, no consistent annual spatio-temporal pattern for cluster emergence and epidemic spread could be observed, thus precluding the capacity to predict where the next epidemic would break out, and what geographical direction it would follow. These findings rely on laboratory-based data and have important public health implications as discussed hereafter.

The first asset of this study was the quality of the microbiological data. We used laboratory-confirmed *N. meningitidis* cases data, coming from a surveillance system managed by CERMES and DSSRE throughout the country. Most other spatio-temporal studies on meningitis epidemics in sub-Saharan Africa [6]–[12], [19] are based instead on suspected cases reported in the framework of the national surveillance systems. In our dataset, none of the three typical bacterial aetiologies (*N. meningitidis*, *S. pneumoniae* and *H. influenzae*) could be identified in almost 60% of the CSF analysed by CERMES over the study period (see Table 1). Relying only on suspected cases would therefore introduce a large number of misclassified cases. However, our system may suffer from underreporting from areas where performing a lumbar puncture and shipping the samples to CERMES may represent logistical difficulties. Further analyses (not shown here) have documented that indeed the districts the most remote from CERMES (in Maradi and Zinder regions) were sending less CSF specimens than the closer ones, for a similar number of suspected cases notified to DSSRE. However, the proportion of negative cases among the received CSF specimens was fairly similar among the healthcare centres (outside the capital Niamey). This suggested that the decision to take or not a CSF sample from a patient based on clinical criteria had no significant spatial variability. Moreover, our cluster analyses enabled us to detect the importance of remote regions in the epidemic dynamics according to the recurrent clusters identified there. Like in many other settings, the surveillance system may not cover the entire population of Niger affected by meningitis. However, we can reasonably assume that most meningitis cases, because of their severity, end up reaching the healthcare centres, with or without prior self-treatment or consultation of a tradi-practitioner. Moreover, free healthcare offered to all people suffering from meningitis in Niger probably reduces social and spatial disparities in care-seeking behaviours. Thus, for all the reasons above, we are confident that the surveillance system is representative enough and that underreporting did not substantially affect the validity of our results, which are more likely to reflect the dynamism peculiar to meningitis than the spatial disparities in the surveillance system efficiency. Incidence estimates were based on the 2001 census and constant population growth rates were applied. We could not take into account possible variations of population growth rate over time and space, due to the difficulty in quantifying population migrations.

The second asset of this study was the use of HCCAs as spatial units for the spatio-temporal analysis of MM. They represent a more accurate spatial unit of analysis than the district level on which reactive vaccination strategies and spatio-temporal studies are usually based [3], [6], [7], [19]. Analysing data at the HCCA level has greater relevance for understanding the epidemic dynamics, for making decisions in response to starting epidemics and for assessing control strategies.

Indeed, this study has shown that clusters most often included only a few HCCAs within a district. This finding, previously suggested by [20], is important for understanding meningitis epidemics and should encourage surveillance at the health centre level. Clusters occurred in different HCCAs within the same districts in consecutive years, demonstrating strong intra-district heterogeneity and year-to-year variability of the affected HCCAs. This could result from outbreaks limited to HCCAs without exceeding the threshold at the district level: the district is not vaccinated and may be affected by a large outbreak the following year. Besides, waiting for the threshold to be reached at the district level to initiate reactive vaccination may incur unnecessary delays: we showed that a decision based on threshold estimated at the health centre level might lead to earlier detection of outbreaks, so more reactive and possibly more cost-effective vaccination strategies. Thus, adding HCCA-level surveillance to the current district-level surveillance would improve the timeliness of epidemic detection.

With the introduction of a new meningococcal A conjugate vaccine (MenAfriVac™) in the meningitis belt over the next few years, the use of the health centre catchment areas as spatial units can also help to monitor more accurately the vaccine supply at a finer spatial scale, saving doses that could be given inadequately, and to evaluate its impact and protective efficacy in the population (herd immunity) at the same level. Although this vaccine brings new hope to the control of meningitis epidemics, reactive vaccination with polysaccharide vaccines and research to improve control strategies will still be needed in the coming years, since it will take several years to immunize against the A the vulnerable population across the belt and since other serogroups like W135 may replace meningitis A as the dominant serogroup [21]. New decision criteria will have to be found for reactive vaccination. With the additional use of a finer spatial scale like the HCCAs, an interesting strategy would be real-time cluster detection, with prospective space-time scan statistic [22] or other existing methods [23].

In the context of a resource-limited country, this study can also assist public health authorities in their decision-making regarding resource allocation. The spatial clusters detected in our study were located in different HCCAs from year to year, but nine of the 42 districts were more recurrently affected by clustering of MM cases. Thus, these findings provide approaches to better adjust allocation of resources, including a ready supply of antibiotics and rapid diagnostic tests [24], [25], as well as additional health care personnel. In order to reduce the reaction time of the vaccination, one may consider allocating vaccines to these districts' hospitals prior to the meningitis season, provided the cold chain can be maintained. Given cost and organizational constraints, further cost-effectiveness and feasibility analyses are needed to evaluate this strategy, before any policy recommendation.

Clusters were more often found in nine districts, including five bordering Nigeria within a 500 km distance between Doutchi and Aguie, most likely because of intense mobility of border populations [26]. However, no consistent annual spatio-temporal pattern could be found over the study period; hence, no spread in a systematic geographical direction from a fixed source could be identified. This is contrary to a study carried out in Mali, which highlighted a potential south-north spread, with Bamako and Mopti as probable sources [12]. Instead, our results suggest the emergence of scattered sources, likely from a pool of carriers when conditions are favorable to the occurrence of the invasive disease. Favorable conditions may include climatic conditions occurring during the dry season (low absolute humidity and dust-laden Harmattan wind), which would damage the nasopharyngeal mucous membrane and increase the risk of bloodstream invasion by a colonizing meningococcus [27]. In this study, we observed that the latest spatio-temporal clusters during the epidemic season were often the northernmost ones, which could be correlated with the northward advance of the Intertropical Front preceding the arrival of rains from the south, thus raising relative humidity. However, climatic factors do not entirely explain these spatio-temporal epidemic patterns. As suggested by Mueller's hypothetical explanatory model [20], their role may be limited to the hyperendemic increase during the dry season, while transition from a hyperendemic state to highly localized epidemics may be due to increased transmission, possibly caused by viral respiratory co-infections. Moreover, in equivalent climatic conditions, an area in which the proportion of susceptible individuals is higher due to waning immunity (acquired by infection or vaccination) would be more prone to outbreaks [28]. Recently, Irving et al [29] suggested that population immunity may be a key factor in causing the unusual epidemiology of meningitis in the Belt. Although density and distance to primary roads were not individually correlated with MM incidence at the HCCA level, other socio-demographic factors (poverty, overcrowded housing, migrations, markets…) may also have an influence on local transmission of the bacteria and carriage and contribute to the risk of micro-epidemics of co-infections [20]. Of note, one spatio-temporal cluster of four adult cases was detected in February 2009 in Bilma district (see figure 1), in the oasis town of Dirkou, located on an important south-north route of trans-Saharan trade and transit migration. Meningococcal strain variations most likely play a role in the occurrence of epidemic waves [20], [30], [31]. In this study, the spatio-temporal distribution of all *N. meningitidis* cases was analysed irrespective of the serogroups. A subsequent analysis will differentiate serogroups of meningococci as their spatio-temporal patterns may significantly vary [32], [33]. Further etiologic studies are needed to explore causality of the spatio-temporal patterns highlighted in this paper.

Finally, our findings provide an evidence-based approach to reflect on public health policies and indicate a promising strategy to improve prevention and control of meningitis in sub-Saharan Africa. They can serve as an example for other meningitis belt countries, illustrating what finer scale surveillance and spatial analyses can offer for prevention and control of meningitis. Research efforts should now focus on investigating the role of dust, socio-demographic factors, co-infections and vaccination strategies on cluster occurrence at the HCCA level, and on developing an operational decision support tool to respond better to meningitis outbreaks with the introduction of the new conjugate vaccine.

## Supporting Information

Text S1 **Supporting information on Global Moran's** ***I*** **, Anselin's Local Moran's** ***I*** **and Kulldorff's spatial scan statistics.**
(DOC)

Checklist S1 **STROBE checklist.**
(DOC)

## References

[1] Lapeyssonnie L (1963). La méningite cérébrospinale en Afrique.. Bull World Health Organ.

[2] World Health Organization (1998). Control of epidemic meningococcal disease. WHO Practical Guidelines. 2nd ed.

[3] World Health Organization (2000). Detecting meningococcal meningitis epidemics in highly-endemic African countries.. Wkly Epidemiol Rec.

[4] Sugumaran R, Larson SR, DeGroote JP (2009). Spatio-temporal cluster analysis of county-based human West Nile virus incidence in the continental United States.. Int J Health Geogr.

[5] Moore DA, Carpenter TE (1999). Spatial analytical methods and geographic information systems: use in health research and epidemiology.. Epidemiol Rev.

[6] Thomson MC, Molesworth AM, Djingarey MH, Yameogo KR, Belanger F (2006). Potential of environmental models to predict meningitis epidemics in Africa.. Trop Med Int Health.

[7] Molesworth AM, Cuevas LE, Connor SJ, Morse AP, Thomson MC (2003). Environmental risk and meningitis epidemics in Africa.. Emerging Infect Dis.

[8] Yaka P, Sultan B, Broutin H, Janicot S, Philippon S (2008). Relationships between climate and year-to-year variability in meningitis outbreaks: a case study in Burkina Faso and Niger.. Int J Health Geogr.

[9] Broutin H, Philippon S, Constantin de Magny G, Courel M-F, Sultan B (2007). Comparative study of meningitis dynamics across nine African countries: a global perspective.. Int J Health Geogr.

[10] Sultan B, Labadi K, Guégan J-F, Janicot S (2005). Climate drives the meningitis epidemics onset in west Africa.. PLoS Med.

[11] Jackou-Boulama M, Michel R, Ollivier L, Meynard JB, Nicolas P (2005). Corrélation entre la pluviométrie et la méningite à méningocoque au Niger.. Med Trop.

[12] Philippon S, Broutin H, Constantin de Magny G, Toure K, Diakite CH (2009). Meningococcal meningitis in Mali: a long-term study of persistence and spread.. Int J Infect Dis.

[13] Sidikou F, Djibo S, Taha MK, Alonso JM, Djibo A (2003). Polymerase chain reaction assay and bacterial meningitis surveillance in remote areas, Niger.. Emerg Infect Dis.

[14] Chanteau S, Sidikou F, Djibo S, Moussa A, Mindadou H (2006). Scaling up of PCR-based surveillance of bacterial meningitis in the African meningitis belt: indisputable benefits of multiplex PCR assay in Niger.. Trans R Soc Trop Med Hyg.

[15] World Health Organization (1999). Laboratory methods for the diagnosis of meningitis caused by Neisseria meningitidis, Streptococcus pneumoniae, and Haemophilus influenzae.

[16] Anselin L (1995). Local indicators of spatial association.. Geographical analysis.

[17] Kulldorff M (1997). A spatial scan statistic.. Commun Statist – Theory Meth.

[18] Kulldorff M, Athas WF, Feurer EJ, Miller BA, Key CR (1998). Evaluating cluster alarms: a space-time scan statistic and brain cancer in Los Alamos, New Mexico.. Am J Public Health.

[19] Cuevas LE, Savory EC, Hart CA, Thomson MC, Yassin MA (2007). Effect of reactive vaccination on meningitis epidemics in Southern Ethiopia.. J Infect.

[20] Mueller JE, Gessner BD (2010). A hypothetical explanatory model for meningococcal meningitis in the African meningitis belt.. Int J Infect Dis.

[21] The Lancet Infectious Diseases (2011). A vaccine against meningitis in Africa.. Lancet Infect Dis.

[22] Kulldorff M (2001). Prospective time periodic geographical disease surveillance using a scan statistic.. J R Stat Soc Ser A Stat Soc.

[23] Watkins R, Eagleson S, Hall R, Dailey L, Plant A (2006). Approaches to the evaluation of outbreak detection methods.. BMC Public Health.

[24] Chanteau S, Dartevelle S, Mahamane AE, Djibo S, Boisier P (2006). New rapid diagnostic tests for Neisseria meningitidis serogroups A, W135, C, and Y.. PLoS Med.

[25] Hamidou AA, Djibo S, Mamane AE, Moussa A, Chanteau S (2008). Serogrouping of non-interpretable Neisseria meningitidis carriage strains, using rapid diagnostic tests.. Trans R Soc Trop Med Hyg.

[26] Maïnassara HB, Molinari N, Dematteï C, Fabbro-Peray P (2010). The relative risk of spatial cluster occurrence and spatio-temporal evolution of meningococcal disease in Niger.. Geospat Health.

[27] Greenwood B (1999). Manson Lecture. Meningococcal meningitis in Africa.. Trans R Soc Trop Med Hyg.

[28] Pascual M, Dobson A (2005). Seasonal patterns of infectious diseases.. PLoS Med.

[29] Irving TJ, Blyuss KB, Colijn C, Trotter CL (2011). Modelling meningococcal meningitis in the African meningitis belt.. Epidemiol Infect.

[30] Taha M-K, Deghmane A-E, Antignac A, Zarantonelli ML, Larribe M (2002). The duality of virulence and transmissibility in Neisseria meningitidis.. Trends Microbiol.

[31] Leimkugel J, Hodgson A, Forgor AA, Pflüger V, Dangy J-P (2007). Clonal waves of Neisseria colonisation and disease in the African meningitis belt: eight-year longitudinal study in northern Ghana.. PLoS Med.

[32] Boisier P, Djibo S, Sidikou F, Mindadou H, Kairo KK (2005). Epidemiological patterns of meningococcal meningitis in Niger in 2003 and 2004: under the threat of N. meningitidis serogroup W135.. Trop Med Int Health.

[33] Boisier P, Nicolas P, Djibo S, Taha M, Jeanne I (2007). Meningococcal Meningitis: Unprecedented Incidence of Serogroup X–Related Cases in 2006 in Niger.. Clin Infect Dis.

