# Supplementary material for: Analysing Spatio-Temporal Clustering of Meningococcal Meningitis Outbreaks in Niger Reveals Opportunities for Improved Disease Control
Source: PLoS Negl Trop Dis. 2012 Mar 20;6(3):e1577. doi: 10.1371/journal.pntd.0001577 (PMC3308932; doi:10.1371/journal.pntd.0001577)
Supplement: Text S1 — Supporting information on Global Moran's I , Anselin's Local Moran's I and Kulldorff's spatial scan statistics. (DOC) [file pntd.0001577.s001.doc]

**Text S1.** Supporting information on Global Moran’s *I*, Anselin’s Local Moran’s *I* and Kulldorff’s spatial scan statistics.

**Global Moran’s *I* statistic**

Spatial autocorrelation is the correlation of a variable with itself across geographic space. Positive spatial autocorrelation indicates that similar values are spatially clustered while negative spatial autocorrelation indicates that nearby places tend to have more dissimilar values than expected by chance, suggesting a spatial pattern similar to a checkerboard. Global autocorrelation statistics provide a single measure of spatial autocorrelation for an attribute in a region as a whole.

The Global Moran’s *I* statistic for MM annual incidence in Niger was given by:

with *N* the total number of health centre catchment areas (HCCAs) in Niger, *X_i_* the annual incidence of meningococcal meningitis (MM) cases in the HCCA *i*, *X_j_* the annual incidence of MM cases in the HCCA *j*, $\overline{X}$ the mean annual incidence for Niger and *W_ij_* a spatial weight measuring the closeness of areas *i* and *j*. *W_ij_* was an inverse function of the distance *d_ij_* between the centroids of two HCCAs *i* and *j*:

$$W_{ij}=\frac{1}{d_{ij}}$$

A threshold distance *δ*  was defined such as when *d_ij_* >*δ*, then *W_ij_ = 0*. Thus, it was considered that two HCCAs would have an influence on each other (and their couple would contribute to the calculation of the Global Moran’s *I*) only if the distance between their centroids was inferior to this defined threshold distance and that this influence decreased with the distance.

To measure the distance where spatial effects were maximized, Global Moran tests were carried out for each year using different threshold distances *δ* with the Spatial Autocorrelation (Moran’s I) tool in ArcGIS Spatial Statistics toolbox (version 9.3, ESRI Inc. Redlands, CA). A range of threshold distances was investigated from 20 km (the mean distance between the centroids of two neighboring HCCAs) to 200 km. Correlograms *I = f(δ)* were plotted in R (version 2.9.1, R Development Core Team, R Foundation for Statistical Computing, Vienna, Austria) and the 95 % confidence interval of the null hypothesis around the expected value was computed at each distance, to identify the distance at which autocorrelation was the most significant for each year. This optimal distance for cluster detection was used to define the distance parameter of both cluster detection methods described below.

**Anselin’s Local Moran’s *I* statistic**

The Anselin’s Local Moran statistics (Local Indicators of Spatial Association or LISA) [13,14] allowed for the decomposition of the Global Moran’s *I* into the contribution of each individual observation. It provided a measure of the spatial autocorrelation for a given HCCA with its neighbours. The null hypothesis was that there was no association between MM incidence rates in nearby HCCAs (no spatial autocorrelation). The alternative hypothesis was that spatial clustering existed (nearby HCCAs did have similar MM incidence rates).

The Local Moran’s *I* in a HCCA *i* was given by:

with *X_i_* the annual incidence of MM cases in the HCCA *i*, *X_j_* the annual incidence of MM cases in the HCCA *j*, $\overline{X}$ the mean annual incidence of MM cases for Niger and *W_ij_* the spatial weight matrix previously defined as the inverse function of the distance *d_ij_* between the centroids of two HCCAs *i* and *j* when *d_ij_* is less than the threshold distance *δ*, null otherwise.

The Cluster and Outlier Analysis tool in ArcGIS Spatial Statistics toolbox was used to generate Anselin’s Local Moran statistics. For each year, the threshold distance was set at the optimal scale previously found with the correlograms of Global Moran’s *I*. The statistical significance was assessed by Z Scores with an alpha level of 5 %. A statistically significant positive value of the Local Moran’s *I* for a HCCA indicated that the surrounding HCCAs had similar incidence rates (IR). It could be either a HCCA with a high IR in a neighbourhood that had high IR too (area described as *high-high*) or a HCCA with a low IR in a neighbourhood that had low IR too (area described as *low-low*). A statistically significant negative value of the Local Moran’s *I* for a HCCA indicated that this HCCA had a different IR from its neighbours. It could be either a HCCA with a high IR in a neighbourhood that had low IR (*high-low* area) or a HCCA with a low IR in a neighbourhood that had high IR (*low-high* area). As the point was to identify high-risk areas, only *high-high* and *high-low* HCCAs were mapped.

**Kulldorff’s spatial scan statistic**

The Kulldorff’s spatial scan statistic was used in the SaTScan software (version 8.0, Kulldorff and Information Management Services, Inc.) to test whether MM cases were distributed randomly over space during each year, and if not, to statistically identify significant clusters of cases [13,15]. This method used a circular window which moved throughout the study area. This window was centred on the centroid of each spatial unit and its radius varies from 0 to a user-defined upper limit (the maximum spatial cluster size). For each location and size of the scanning window, the software calculated a likelihood ratio function to test for elevated risk within the window as compared to outside. The null hypothesis was that the risk was the same throughout the study area. The window with the maximum likelihood was defined as the “most likely” cluster (i.e. the cluster least likely to have occurred by chance). The programme also identified secondary clusters and ordered them according to their likelihood ratio test statistic. A cluster of disease cases could be constituted by either a high-risk single HCCA or a high-risk group of HCCAs.

The analyses were conducted for each year separately like in the first method. A data file containing MM cases and population data for the centroid (X and Y coordinates) of each healthcare centre area in Niger was imported into SaTScan assuming a Poisson probability disease model (case and at-risk population data). The maximum spatial cluster size was set at the optimum distance identified with the correlograms of Global Moran’s *I*. Only non-overlapping high-risk clusters were reported. *P*-values for maximum likelihood ratios were based on 999 Monte Carlo replications of the data set. An alpha level of 5 % was used to assess statistical significance.

To introduce the temporal component and test whether MM cases were distributed randomly over space and time, a space-time scan [17] was also performed, by defining a cylindrical window with a circular geographic base and with height corresponding to time. The radius of the base was varying between zero and the same maximum spatial cluster size as in the purely spatial scan, while the height of the cylinder, reflecting the time period of the potential clusters, was varying between zero and one week. This maximum temporal cluster size of one week was chosen to be consistent with the epidemiological surveillance based on weekly counts of cases.
